# Dissolution Enhancement and Controlled Release of Paclitaxel Drug via a Hybrid Nanocarrier Based on mPEG-PCL Amphiphilic Copolymer and Fe-BTC Porous Metal-Organic Framework

**DOI:** 10.3390/nano10122490

**Published:** 2020-12-11

**Authors:** Nikolaos D. Bikiaris, Nina Maria Ainali, Evi Christodoulou, Margaritis Kostoglou, Thomas Kehagias, Emilia Papasouli, Emmanuel N. Koukaras, Stavroula G. Nanaki

**Affiliations:** 1 Laboratory of Chemistry and Technology of Polymers and Dyes, Department of Chemistry, Aristotle University of Thessaloniki, GR-541 24 Thessaloniki, Greece; nbikiaris@gmail.com (N.D.B.); ainali.nina@gmail.com (N.M.A.); evicius@gmail.com (E.C.); 2 Laboratory of General and Inorganic Chemical Technology, Department of Chemistry, Aristotle University of Thessaloniki, GR-541 24 Thessaloniki, Greece; kostoglu@chem.auth.gr; 3 Laboratory of Electron Microscopy, Department of Physics, Aristotle University of Thessaloniki, GR-541 24 Thessaloniki, Greece; kehagias@auth.gr; 4 Laboratory of Quantum and Computational Chemistry, Department of Chemistry, Aristotle University of Thessaloniki, GR-541 24 Thessaloniki, Greece; aimipapa@chem.auth.gr (E.P.); koukarase@chem.auth.gr (E.N.K.)

**Keywords:** mPEG-PCL copolymer, Fe-BTC, metal-organic framework, paclitaxel, nanoparticles, dissolution enhancement, controlled release, quantum chemistry, tight binding

## Abstract

In the present work, the porous metal-organic framework (MOF) Basolite^®^F300 (Fe-BTC) was tested as a potential drug-releasing depot to enhance the solubility of the anticancer drug paclitaxel (PTX) and to prepare controlled release formulations after its encapsulation in amphiphilic methoxy poly(ethylene glycol)-poly(ε-caprolactone) (mPEG-PCL) nanoparticles. Investigation revealed that drug adsorption in Fe-BTC reached approximately 40%, a relatively high level, and also led to an overall drug amorphization as confirmed by differential scanning calorimetry (DSC) and X-ray diffraction (XRD). The dissolution rate of PTX-loaded MOF was substantially enhanced achieving a complete (100%) release within four days, while the neat drug only reached a 13% maximum rate (3–4 days). This PTX-Fe-BTC nanocomposite was further encapsulated into a mPEG-PCL matrix, a typical aliphatic amphiphilic copolyester synthesized in our lab, whose biocompatibility was validated by in vitro cytotoxicity tests toward human umbilical vein endothelial cells (HUVEC). Encapsulation was performed according to the solid-in-oil-in-water emulsion/solvent evaporation technique, resulting in nanoparticles of about 143 nm, slightly larger of those prepared without the pre-adsorption of PTX on Fe-BTC (138 nm, respectively). Transmission electron microscopy (TEM) imaging revealed that spherical nanoparticles with embedded PTX-loaded Fe-BTC nanoparticles were indeed fabricated, with sizes ranging from 80 to 150 nm. Regions of the composite Fe-BTC-PTX system in the infrared (IR) spectrum are identified as signatures of the drug-MOF interaction. The dissolution profiles of all nanoparticles showed an initial burst release, attributed to the drug amount located at the nanoparticles surface or close to it, followed by a steadily and controlled release. This is corroborated by computational analysis that reveals that PTX attaches effectively to Fe-BTC building blocks, but its relatively large size limits diffusion through crystalline regions of Fe-BTC. The dissolution behaviour can be described through a bimodal diffusivity model. The nanoparticles studied could serve as potential chemotherapeutic candidates for PTX delivery.

## 1. Introduction

Cancer is a term describing the disease resulting when cellular changes cause the uncontrolled growth and division of cells. There are types of cancer causing rapid cell growth, while others cause cells to grow and divide at a slower rate [1]. Chemotherapy aims to kill cancerous cells with medications that target rapidly dividing cells. Paclitaxel (PTX) is a well-known chemotherapeutic agent used in the treatment of various cancer types including breast, lung, ovarian, bladder, and prostate cancer [2]. However, its low solubility remains an obstacle for its delivery. So far, many systems for the enhanced delivery of paclitaxel are proposed. Nanoparticles are widely used in biomedical applications, either as drug-delivery systems, as drug solubility enhancers, or for magnetic resonance imaging (MRI) [3]. They can be used either neat or modified, incorporated into biocompatible and biodegradable polymers using various techniques such as single or double emulsification methods, ionic gelation, electrospinning, 3D printing and others. Nanoparticle delivery systems are the most promising ones in cancer therapy due to their ability to be guided to the tumor areas under certain circumstances, their tumor-penetrating capability, and the enhanced cellular uptake leading to a more efficient drug delivery. Biocompatible aliphatic polyesters have been extensively used for the nanoencapsulation of PTX, providing a controlled release behaviour. This is crucial in the case of PTX formulations, in order to prolong drug administration for a desired period and also minimise side effects and chances of toxicity caused by the immediate one-time administration of high drug doses.

Obayemi et al. [4] used typical aliphatic polyesters; i.e., poly(caprolactone) (PCL), poly(lactic-co-glycolic acid) (PLGA), poly(ethylene glycol) (PEG) and their blends for transdermal delivery of paclitaxel via 3D porous films. Our lab has previously implemented several strategies to develop formulations for paclitaxel delivery. At a glance, Nanaki et al. [5] prepared and studied paclitaxel-loaded PLGA microparticles for local chemotherapy, where PTX had previously been incorporated into mesoporous silica nanoparticles (SBA-15). It was found that the adsorption of paclitaxel in SBA-15 enhanced and prolonged the delivery of the drug. Christodoulou et al. [6] synthesized thioether-containing ω-hydroxyacid-co-poly(d,l-lactic acid) (TEHA-co-PDLLA) semitelechelic block copolymers in various ratios, then used them to prepare nanoparticles loaded with PTX and manganese ferrite magnetic nanoparticles (MnFe_2_O_4_ MNPs) for a combined hyperthermia and chemotherapy treatment. Filippousi et al. [7] used novel nanoscale metal-organic frameworks (MOFs) for the adsorption of paclitaxel and further encapsulated the nanocomposite into poly(ε-caprolactone) (PCL)-b-TPGS microparticles. It was found that the drug release was depended on the interaction between the MOFs and the drug, while the controlled release rates were attributed to the microencapsulation. Karavelidis et al. [8] synthesized novel poly(ethylene glycol) methyl ether-poly(propylene adipate) (mPEG-PPAd) copolymers for the nanoencapsulation of paclitaxel, developing a pH- and thermo-sensitive nanosystem for cancer treatment with improved drug release at 42 °C and pH = 6.

Recent trends in the field deal with the co-delivery of anti-cancer drugs and/or inhibitors to further increase the therapeutic effect. Liu et al. [9] proposed a co-delivery system of paclitaxel and quercetin, a P-gp inhibitor, via their incorporation in modified silica nanoparticles showing enhanced targeting, prolonged tumor retention time and effective anti-tumor effect without obvious toxicity to normal tissues in vivo. Gao et al. [10] proposed a co-delivery of paclitaxel and curcumin via PEGylated lipid bilayer coated mesoporous silica nanoparticles. It was found that the proposed formulation effectively carried the drugs into cancer cells, mainly localized in lysosomes and mitochondria, with sustained release characteristics. Li et al. [11] proposed the co-delivery of doxorubicin (DOX) and PTX by reduction/pH dual-responsive nanocarriers. They synthesized PEGylated poly(a-lipoic acid) copolymer (mPEG-PaLA) and used it for a co-loaded nanoparticles formulation. Nanoparticles showed enhanced PTX and DOX release in response to reductive and acidic stimuli, while drugs were released intracellularly to osteosarcoma cells. Uthappa et al. [12] recently published a review article concerning the use of nanodiamonds (NDs) in drug-delivery systems. Their surface modifications enhance their properties and render them promising in drug-delivery systems. Lim et al. [13] designed stable and colloidal dispersity, the carboxylated NDs (COOH-NDs) reduced to hydroxylated NDs (OH-NDs). PTX was loaded to OH-NDs and showed sustained release profile for up to 70 h with value reached at 97.3%. Bazzazzadeh et al. [14] proposed the magnetic MIL-53 nanometal organic framework particles (NMOFs) incorporated into poly(acrylic acid)-grafted chitosan/polyurethane (PA-g-CS/PU) core-shell nanofibers for controlled release of temozolomide (TMZ) and PTX against U-87 MG glioblastoma cells during chemotherapy/hyperthermia combination therapy. They managed to deliver PTX to specific regions with a maximum apoptosis of 49.6% of U-87 MG glioblastoma cells under AMF. Electro-spinning has also been used by Mehnath et al. [15] in biodegradable nanofibers preparation containing paclitaxel as ligand coupled to micelles. The aim was to prepare a pH-responsive and tumor-targeting drug-delivery formulation.

MOFs have been used widely in the last decade as potential drug-carrier nanovehicles, due to their significant opportunity to tune their pore size and their ease to adjust the framework’s functional groups [7,16,17,18,19,20]. Fe-BTC is a new porous MOF consisting of Fe metal ions and 1,3,5-benzenetricarboxylate (BTC) that has already been used in water dye purification systems [21], in metal recovery from water wastes [22,23], in enzyme immobilization [24] and also as a catalyst [25]. Its multifunctional use is a consequence of its high porosity values. Concerning drug delivery, there is only one study found in literature [26], where Adhikari et al. modified Fe-BTC with mesoporous silica nanoparticles (MSN) in order to develop a hybrid drug-delivery system and prevent premature drug release from bare-MSN. Fe-BTC has a high specific area (1300–1600 m^2^/g) and could be an ideal matrix for drug-dissolution enhancement.

The aim of the present study was to enhance the dissolution rate of PTX and also to prepare sustained and controlled release formulations. To achieve both, we propose the use of Fe-BTC for paclitaxel adsorption and its further incorporation onto mPEG-PCL nanoparticles. mPEG-PCL is a typical aliphatic polyester, both biocompatible and biodegradable, widely used in drug delivery systems. To the best of our knowledge, no similar studies have previously been conducted. 

## 2. Materials and Methods

### 2.1. Materials

Poly(ethylene glycol) methyl ether (mPEG; average Mn 750 g/mol), ε-caprolactone (ε-CL; purum 99%), stannous octoate (Sn(Oct)_2_; purum 95.0%), Sodium cholate hydrate (purum ≥ 99%) and Basolite^@^Fe300 (Fe-BTC; Mw = 262.96) were purchased from Sigma-Aldrich (Darmstadt, Germany). ε-CL was double distilled at 180 °C under reduced pressure at about 5 mmHg prior to use in order to remove the inhibitor contained within. Paclitaxel (purum 99.5%) was kindly donated by Pharmathen S.A. (Athens, Greece). All other chemical sand reagents were of analytical grade.

### 2.2. Synthesis and Characterization of Poly(Ethylene Glycol) Methyl-Poly(Caprolactone) (mPEG-PCL)

#### 2.2.1. Synthesis of mPEG-PCL

The synthesis of mPEG-PCL was performed by ring opening polymerization as previously described [8,27]. Briefly, accurately weighed amounts of mPEG and ε-CL were mixed together at weight ratio (*w*/*w*) of 30/70 and were placed in a round-bottom flask that was equipped with a mechanical stirrer, condenser, and nitrogen inlet. A proper amount of stannous octoate catalyst was also added. The polymerization mixture was de-gassed and purged with nitrogen several times, inserted into a heated salt bath at 180 °C, and kept there under constant stirring (550 rpm) and nitrogen atmosphere over a period of 1.5 h. After that, a vacuum was applied to remove any unreacted ε-CL for 10 min. The reaction flask was then quenched to room temperature (RT), and the products were purified by dissolving in chloroform and precipitated in cold methanol three times before usage. The precipitate was filtered and dried in a vacuum oven at 35 °C for 24 h.

#### 2.2.2. Characterization of mPEG-PCL

Nuclear magnetic resonance (^1^H-NMR) spectra of mPEG-PCL were obtained by means of an Agilent spectrometer (Agilent Technologies, Santa Clara, CA, USA) operating at a frequency of 500 MHz for protons at RT. Deuterated cholorofom (CDCl3) was used as solvent to prepare solutions of 5% *w*/*v*. Spectra were internally referenced with tetramethylsilane (TMS) and calibrated using the residual solvent peak. The number of scans was 16 for proton and 64 for carbon spectra, and the sweep width was 6 kHz.

Intrinsic viscosity measurements on the isolated polymers were performed using an Ubbelohde capillary viscometer at 25 °C in chloroform at a solution concentration of 1 wt%. The polymer was dissolved in chloroform at final concentration 1% *w/v* and filtered through a disposable membrane filter (0.2 mm, Teflon). Intrinsic viscosity was calculated using the Solomon–Ciuta equation [28]:

[η] = [2(*t*/*t*_0_ − ln(*t*/*t*_0_) − 1)]^1/2^/*c* (1)

where, *c* is the concentration of the solution, *t* the flow time of solution, and *t*_0_ the flow time of pure solvent.

Βased on the measured intrinsic viscosity values the copolymer viscosity-average molecular weight was estimated using the Mark–Houwink–Sakurada (MHS) equation:

[η] = *kM_v_^a^* (2)

where, *Mν* is the viscosity-average molecular weight, and *k* and *a*, are the constants for a given solute–solvent system. In the present study, *a* and *k* values were set as 0.6021 and 1.09 × 10^−^^3^ dL g^−^^1^ respectively.

Fourier transform infrared (FT-IR) spectra were obtained using a Perkin Elmer FT-IR spectrometer (model Spectrum One, Perkin Elmer, Dresden, Germany). 18 mg of its sample, fine grounded were mixed with 180 mg KBr and pellets were formed under 5000 tons pressure for 1 min. Spectra were obtained in the region of 400–4000 cm^−1^ using a resolution of 4 cm^−1^ and 32 co-added scans.

A wide angle X-ray scattering (WAXD) study, in the form of thin films, was performed over the range *2θ* from 5° to 60°, at steps of 0.05° and counting time of 5 s, using a MiniFlex II X-ray diffractometry (XRD) system from Rigaku Co. (Chalgrove, Oxford, UK) with CuKa radiation (*λ* = 0.154 nm).

A differential scanning calorimeter (DSC) study was performed by using Pyris Diamond DSC (Perkin Elmer, Dresden, Germany), calibrated with indium and Zinc standards. Samples of 5.0 ± 0.1 mg sealed in aluminum pans were heated up to 250 °C at a heating rate of 20 °C/min under nitrogen atmosphere and remained at that temperature for 1 min. Then the samples were cooled to 25 °C at a cooling rate of 200 °C/min in order to prevent re-crystallization and then re-heated again up to 250 °C with the same heating rate (20 °C/min). From this second DSC scan, the melting point (T_m_) of the paclitaxel was recorded.

For the hydrolytic degradation study, samples of (a) neat mPEG-PCL, and (b) mPEG-PCL containing Fe-BTC were prepared in the form of films (5 × 5 cm and 5 mm thickness) by a PW 30 Otto Weber hydraulic press connected to a temperature controller (Omron E5AX, OMRON Corporation, Kyoto, Japan). The samples were incubated at 37 ± 1 °C for 20 days in Petri dishes containing phosphate buffered saline (PBS) (pH = 7.4). After specific period intervals, the films were removed from the Petri dishes, washed with distilled water and weighed until constant weight. The degree of biodegradation was estimated by mass loss of pre-weighed samples.

*Weight loss* percentage of the polyester films was obtained according to the following equation:

(3) $$Weight\;loss\;\left(\%\right)=\left[\frac{W_{0}-\;W_{r}}{W_{0}}\right]\times 100$$

where $W_{0}$ is the weight of the specimens before degradation, $W_{r}$ the weight of the specimens after hydrolysis and drying.

The in vitro cytotoxicity of the prepared mPEG-PCL copolymers was evaluated in comparison to biocompatible poly(lactic acid) (PLA), by measuring the viability of human umbilical vein endothelial cells (HUVEC) in the presence of different concentrations of the polymers. Cell viability was determined by the 3-(4,5-dimethylthiazol-2-yl)-2,5-diphenyl tetrazolium bromide) (MTT) assay. HUVEC were seeded in 24-well plates at a density of 30,000 cells per well in 500 μL cell culture medium. Twenty-four hours after plating, different amounts of aliphatic polyesters in the form of nanoparticles (suspended in culture medium) were added to the wells. After 24 h of incubation at 37 °C, 50 μL of MTT solution (5 mg/mL in PBS pH 7.4) were added into each well and plates were incubated at 37 °C for 2 h. The intermediate was withdrawn and 200 μL acidified isopropanol (0.33 mL HCl in 100 mL isopropanol) were added in each well and agitated thoroughly to dissolve the crystals formed. The solution was transferred to 96-well plates and immediately read on a microplate reader (Biorad, Hercules, CA, USA), at a wavelength of 490 nm. The experiments were performed in triplicate. Biocompatibility of the polymers was expressed as % cell viability, which was calculated from the ratio between the number of cells treated with the nanoparticles and that of non-treated cells (control).

### 2.3. Nanoparticles Preparation and Characterization

#### 2.3.1. Adsorption of Paclitaxel onto Fe Metal Ions and 1,3,5-Benzenetricarboxylate (Fe-BTC)

We dissolved 50 mg paclitaxel completely in 100 mL of methanol. 50 mg of Fe-BTC were added to the solution and the resulting dispersion was left under magnetic stirring for 24 h. The Fe-BTC with adsorbed paclitaxel (Fe-BTC-PTX) nanocomposite was then isolated by centrifugation at 12,500 rpm for 20 min. The precipitate was collected, washed once with water, in order to remove excess paclitaxel, and freeze-dried.

#### 2.3.2. Nanoparticles Preparation

Polymeric nanoparticles containing Fe-BTC with adsorbed paclitaxel were prepared by the modified solid-in-oil-in-water (s/o/w) double emulsion/solvent evaporation method. According to this procedure, 50 mg mPEG-PCL were dissolved in 5 mL of dichloromethane. 5 mg of Fe-BTC-PTX nanocomposite were inserted in the polymeric solution and dispersed using a probe sonicator for 1 min. The dispersion was added to 20 mL of sodium cholate aqueous solution, 0.1% *w/v* in concentration, sonicated for 1 min and left under magnetic stirring until total evaporation of dichloromethane. Nanoparticles were collected by centrifugation at 9500 rpm for 20 min and washed several times with ultrapure water. The Fe-BTC-PTX nanoparticles aqueous suspension obtained was finally freeze-dried and stored at 4 °C until further use. Nanoparticles containing neat paclitaxel were also prepared for comparative reasons following the same procedure as described above.

#### 2.3.3. Nanoparticles Characterization

Nitrogen adsorption/desorption experiments at −196 °C were performed for the determination of surface area (multi-point BET method), total pore volume (at *P*/*P*_0_ = 0.99), and pore size distribution (BJH method using adsorption data) of the Fe-BTC samples which were previously outgassed at 150 °C for 16 h under 6.6 × 10^−9^ mbar vacuum using an Automatic Volumetric Sorption Analyzer (Autosorb-1MP, Quantachrome, Anton Paar GmbH, Graz, Austria).

The chemical structure of the synthesized materials was determined with the use of FT-IR spectroscopy. FT-IR spectra of the samples were received with an FT-IR spectrometer (model FT-IR-2000, Perkin Elmer) using KBr discs (thickness of 500 μm). Infrared (IR) absorbance spectra were obtained between 450 and 4000 cm^−1^ at a resolution of 4 cm^−1^ using 64 co-added scans. All spectra presented were baseline corrected and normalized.

X-ray powder diffraction (XRD) patterns were recorded using an XRD-diffractometer (Rigaku-MiniFlex II) with a Cu Kα radiation for crystalline phase identification (*λ* = 0.15405 nm for CuKα). The sample was scanned from 5 to 60 °C.

Particle size distribution of the prepared nanoparticles was determined by dynamic light scattering (DLS) using a Zetasizer Nano instrument (ZEN 3600; Malvern Instruments, Malvern, Worcestershire, UK) operating with a 532 nm laser. A suitable amount of nanoparticles was dispersed in distilled water and kept at 37 °C prior to the measurement. For each sample, three measurements were conducted.

The morphological properties of the nanoparticles in all stages were evaluated by transmission electron microscopy (TEM) experiments in a Jeol JEM 2011 and a Jeol JEM 1010 electron microscopes (JEOL Ltd., Tokyo, Japan), operated at 200 kV and 100 kV, respectively. Samples for TEM imaging were prepared by sonication of each material provided in either pure ethanol or distilled water for 1 h, followed by drop casting onto carbon-coated 400 mesh Cu grids and then dried in air.

DSC was performed as described in Section 2.2.2.

#### 2.3.4. In Vitro Drug Release

In vitro release rates of PTX from the prepared formulations were measured in a USP dissolution apparatus I (basket apparatus, DISKTEK 2100 C with an auto sampler DISTEK EVOLUTION 4300 and a DISKTEK syringe pump, Distek Inc., North Brunswick, NJ, USA). Dissolution tests were performed in 500 mL phosphate buffer (pH = 7.4, *T* = 37 ± 0.5 °C). The rotation speed was set at 50 rpm. At predetermined time intervals, 2 mL of the aqueous solution was withdrawn from the release media. The samples were filtered and assayed for drug release by the high-performance liquid chromatography (HPLC) method which was described previously [5]. In each experiment, the samples were analyzed in triplicate.

#### 2.3.5. High-Performance Liquid Chromatography (HPLC) Quantitative Analysis

Quantitative analysis was performed using a Shimadzu HPLC prominence system consisting of a degasser (DGU-20A5), a liquid chromatograph (LC-20 AD), an auto sampler (SIL-20AC), an ultraviolet–visible (UV/Vis) detector (SPD-20A) and a column oven (CTO-20AC) (SHIMADZU CORPORATION, Kyoto, Japan). A previously validated method was used for the analysis (Vasudev et al., 2012). In detail, a C18 reversed-phase column (250 mm × 4.6 mm i.d., 5-μm particle) was used, the mobile phase was methanol–: 0.05 mM ammonium acetate buffer (pH 7) 80:20 (*v*/*v*) and the flow rate was 1 mL min^−1^. UV detection was performed at 250 nm.

The nanoparticle yield (%), drug loading (%) and entrapment efficiency (%) were calculated using the following equations:

(4) $$Nanoparticles\;Yield\;\left(\%\right)=\;\frac{weight\;of\;nanoparticles}{initial\;weight\;of\;polymers\;and\;PTX}\;\times \;100$$

(5) $$Drug\;Loading\;\left(\%\right)=\;\frac{weight\;of\;drug\;in\;nanoparticles}{total\;weight\;of\;nanoparticles}\;\times \;100$$

(6) $$Entrapment\;Efficiency\;\left(\%\right)=\;\frac{weight\;of\;PTX\;in\;nanoparticles}{initial\;weight\;of\;PTX}\;\times \;100$$

### 2.4. Technical Details on the Computation

To obtain an insight into the systems’ substructures and the interaction between system components there is a requirement to balance between employing high accuracy methods while considering the computational costs that render the computations tractable. We employed the GFN1-xTB method, recently developed by Grimme [29,30], that satisfies these conditions and enables computations on extensive and demanding systems that include transition metal atoms. The noteworthy performance of GFN1-xTB on association energies, non-covalently bound systems and transition metal-containing systems [30] renders the method very suitable for the systems under study.

#### 2.4.1. Structural and Vibrational Analysis

All of the structures except the cages have been optimized using the GNF1-xTB method [29,30]. Determining the spin state of these iron-containing structures is a non-trivial task. For catalytic non-related works, emphasis has not been placed on spin assignment or in some cases even ignored [31]. In a recent and perhaps the most thorough work on the spin state of related species, Vitillo et al. [32] employ high-level wavefunction methods and (single-determinant) density functional theory. They found that specifically for Fe_3_ systems within DFT the intermediate spin state is the most stable due to antiferromagnetic coupling, namely a quintet, whereas wavefunction methods reveal a very narrow energy region between the various spin states suggesting the high spin state. The findings of antiferromagnetic coupling are also corroborated experimentally my Mali et al. [33]. We assign to the iron trimers the intermediate quintet spin state and also attain similar results using a triplet state. Geometry convergence criteria were set to tight and specifically to 8 × 10⁻⁴ a.u. for the cartesian gradient (residual forces). The two cage structures are each of considerable size and amount to a total of 2510 atoms for s-cage (including 150 Fe atoms) and 3514 atoms (including 210 Fe atoms) for l-cage. Taking into account the large size of both cage structures, the GFN-FF [34] method was employed for the geometry optimization in those cases.

#### 2.4.2. Interaction Energy

Interaction energies were evaluated between PTX and a modelled basic building block of Fe-BTC. Long-range dispersion corrections are treated by Grimme’s D3 dispersion method that is well established [35], with Beck–Johnson damping [36]. All computations were performed using the xTB package [29]. The Mercury software [37] was used to create images of the molecular structures.

## 3. Results & Discussion

### 3.1. Synthesis and Characterization of mPEG-PCL

**Figure 1:**
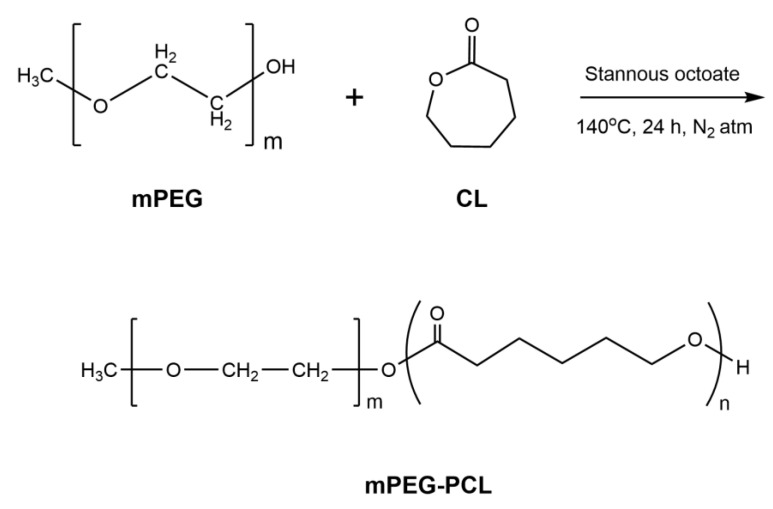
Synthesis of poly(ethylene glycol) methyl-poly(caprolactone) (mPEG-PCL).

mPEG-PCL 30/70 copolymer was synthesized according to the ring opening polymerization procedure described in Section 2.2.1. It is expected that the hydroxyl groups of mPEG will activate stannous octoate and initiate the ring opening polymerisation of ε-CL producing mPEG-PCL block copolymers (Figure 1). Intrinsic viscosity [η] of copolymer was found to be 0.68 dL/g, corresponding to average *Mv* of 43,629 g/dL.

**Figure 2:**
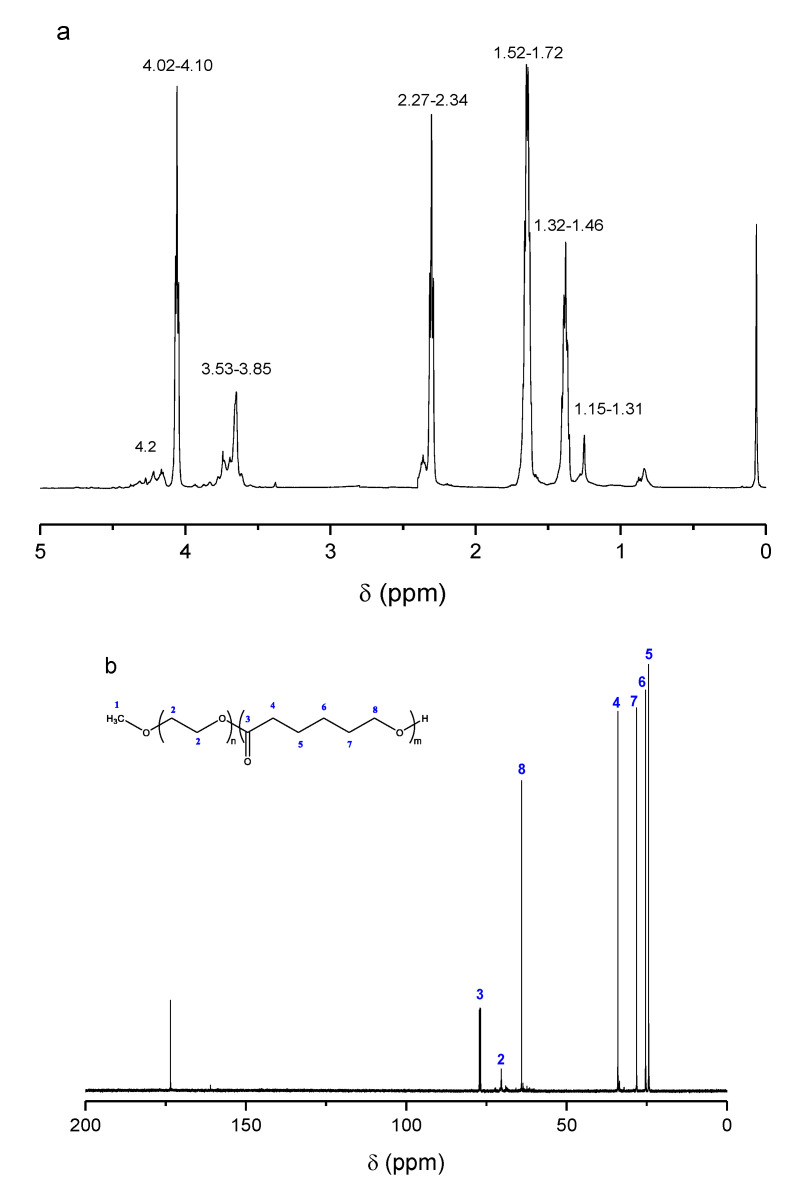
(**a**) Nuclear magnetic resonance (^1^H-NMR) and (**b**) ^13^C-NMR spectra of the synthesized mPEG-PCL copolymer.

^1^H and ^13^C-NMR were used to verify the successful synthesis of mPEG-PCL copolymer. As can be observed in Figure 2a, all characteristic peaks of PCL are present in ^1^H-NMR spectra; i.e., a multiple peak at 1.32–1.46 attributed to methylene group c, a peak at 1.52–1.72 attributed to methylene group b, a triple peak at 2.27–2.34 corresponding to the methylene d next to the carbonyl group and a triple peak at 4.02–4.1 attributed to the methylene group a, standing next to oxygen atoms [27]. Peaks at 3.53 to 3.85 are attributed to the hydrogen atom of methylene groups of mPEG, while the peaks at around 4.2 ppm are attributed to the hydrogen atom of the methylene group next to oxygen bonded to the PCL block [38]. This is a proof that the copolymerisation was successful. Composition of the polymer was calculated using ^1^H-NMR spectra. Mass ratio used in synthesis was mPEG/PCL 30/70 *w*/*w* while calculated weight ratio was found to be 21.69/78.31 *w*/*w*.

In the ^13^C spectrum, the characteristic resonance signal of PCL ester moiety is observed at 173.4 ppm, confirming also the successful polymerization, whereas the small peak at 70.4 ppm is attributed to the equivalent methylene groups of mPEG (CH_2_ 2). All remaining five peaks correspond to the methylene groups of PCL, as follows: 24.5 (CH_2_ 5), 25.4 (CH_2_ 6), 28.2 (CH_2_ 7), 34.0 (CH_2_ 4) και 64.0 (CH_2_ 8) ppm.

**Figure 3:**
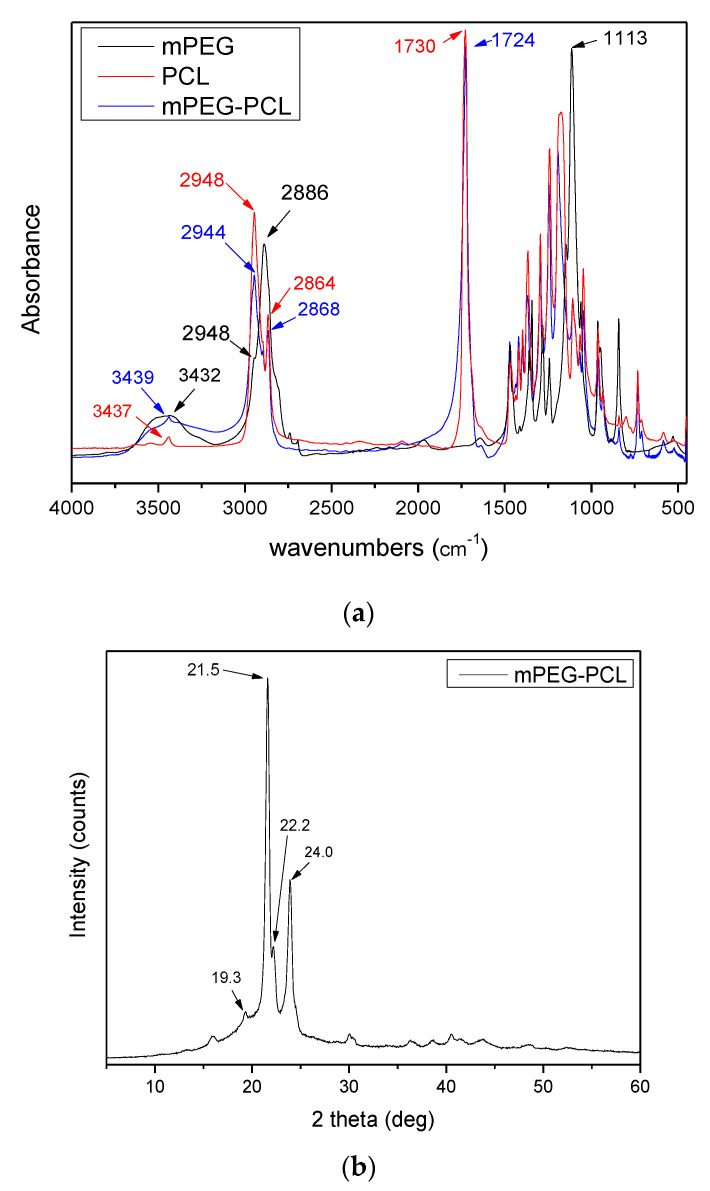
(**a**) Fourier transform infrared (FT-IR) spectra of mPEG, PCL [39] and the synthesized mPEG-PCL copolymer and (**b**) X-ray diffraction (XRD) pattern of mPEG-PCL copolymer.

In addition to NMR, FT-IR spectroscopy was also employed to confirm the structure of the synthesized material (Figure 3a). The spectrum of PCL showed all its characteristic peaks; a peak at 3437 cm^−^^1^ corresponding to –OH groups of PCL, a double peak at 2948 and 2864 cm^−^^1^ corresponding to CH_2_ methylene groups, a peak at 1730 cm^−^^1^ corresponding to C=O and in the region of 1000 to 1500 cm^−^^1^, corresponding to C–C, C–H and C–O groups of the neat PCL. In the spectrum of mPEG, a peak at 3432 cm^–1^ owing to the –OH groups, and two peaks at 2886 cm^−^^1^ and 2948 cm^−^^1^ corresponding to the stretching vibrations of the methylene groups (symmetrical and asymmetrical respectively) were recorded [6] and a strong peak at 1113 cm^−1^ due to C–O absorbance. mPEG-PCL showed all the above characteristic peaks of PCL, i.e., at 3437 cm^−^^1^ (–OH), at 1724 cm^−^^1^ of carboxylic ester (C=O) group and the characteristic peak at 1113 cm^−^^1^ of mPEG, implying that mPEG-PCL copolymer has been successfully prepared.

The XRD pattern of the synthesized mPEG-PCL copolymer revealed a high crystalline structure (Figure 3b). In brief, mainly two crystalline peaks are recorded, i.e., at the 2*θ* positions οf 21.5° and 24° attributed to the PCL blocks. The quite weaker crystalline peaks observed at 19.3° and 22.2° are owing to the mPEG segments of the copolymer [8].

### 3.2. Characterization of Fe-BTC and Fe-BTC with Adsorbed Paclitaxel (Fe-BTC-PTX) Samples

**Figure 4:**
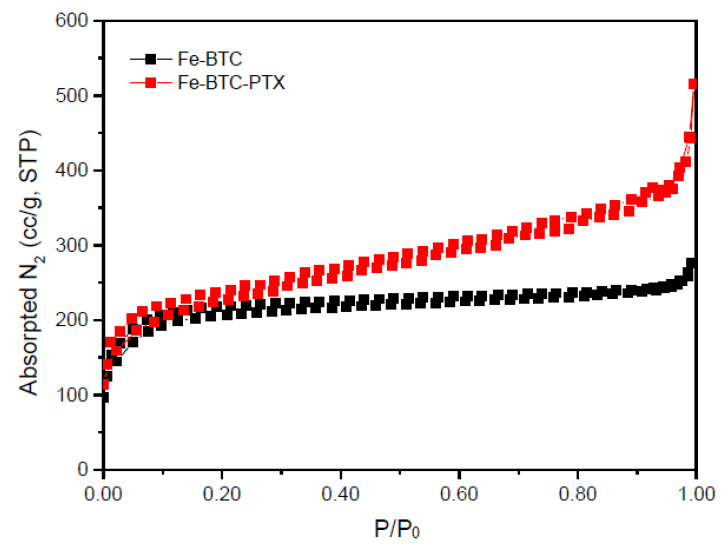
N_2_ adsorption-desorption isotherms.

The porous characteristics of the Fe-BTC before and after paclitaxel adsorption were studied by N_2_ porosimetry at −196 °C and the respective data are presented in Figure 4 and Table 1. The specific surface area (BET method) of Fe-BTC was found to be 1370 m^2^/g, which is in agreement with those reported by the manufacturing company (BET surface area 1300–1600 m^2^/g). A decrease of the value to 826 m^2^/g was observed after the adsorption of paclitaxel drug. The total pore volume also dropped from 0.798 to 0.428 cc/g, probably due to drug absorption by Fe-BTC pores on its surface, and a reduced micropore area and volume was estimated as well, possibly implying the presence of the drug in the micropores of MOF. Similar results have been found in a previous work of ours, where taxol was incorporated in the UiO-66 and UiO-67 MOFs [7]. However, this study cannot provide sufficient evidence to definitively distinguish if the adsorption of PTX occurred mainly in the nanopores or on the surface of Fe-BTC.

HPLC was used to quantify the drug adsorbed on Fe-BTC. In brief, a proper amount of Fe-BTC-PTX was dispersed in 20 mL methanol using probe sonicator for 2 min and was then left under magnetic stirring at RT for 24 h to allow drug release. A significant 40% drug loading in the MOF nanoparticles was established, indicating the high loading capacity of Fe-BTC. This high adsorption rate could also explain the reduced values of surface area and total pore volume that were previously calculated using BET analysis. Such high drug-loading percentages have been also reported in literature for the drug aceclofenac in different types of MOF like MIL100(Fe) [40] and ibuprofen in Cr-MIL-53 and Fe-MIL-53 MOFs [41].

**Figure 5:**
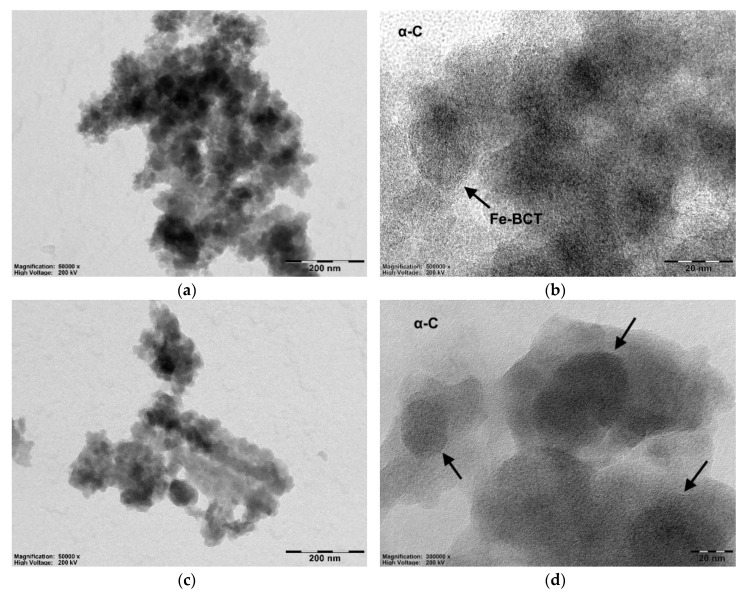
(**a**) Transmission electron microscopy (TEM) micrograph showing an aggregate of amorphous neat Fe-BTC nanoparticles. (**b**) The nanoporous network of Fe-BCT at higher magnification. (**c**) TEM micrograph of an aggregate of neat Fe-BTC after taxol encapsulation. (**d**) Taxol bounded Fe-BTC nanoparticles (arrows), with their nanoporous network still visible.

Although HPLC analysis helped determine the amount of paclitaxel loaded in the Fe-BTC MOF, it could not provide any further insight into how and where exactly the drug was loaded. Therefore, TEM imaging was utilized and the recorded micrographs are presented in Figure 5. As can be seen, neat Fe-BTC MOF appears in the form of quasi-spherical amorphous nanoparticles with average sizes of 20 to 30 nm (Figure 5a), whereby their nanopore network is visible at a higher magnification (Figure 5b), since nanopores exhibit a darker contrast than the surrounding material. After paclitaxel adsorption, the Fe-BTC nanoparticles retain their original sizes (Figure 5c), which implies that they are rather stable, at the used conditions, for drug encapsulation. Moreover, from higher magnification images it appears that nanoparticles are surrounded by amorphous material, which is probably PTX (Figure 5d). This suggests that due to interactions, also visible in the FT-IR spectrum, PTX is readily bounded with Fe-BTC nanoparticles, partially filling their nanopore structure. Unfortunately, due to the amorphous nature of all materials, PTX encapsulation inside the Fe-BTC nanopores cannot be clearly distinguished.

**Figure 6:**
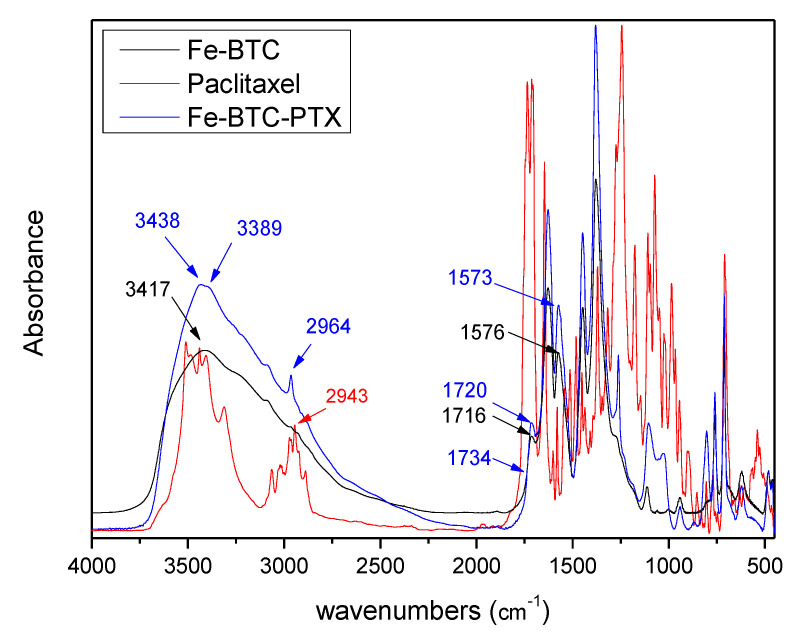
Fourier transform infrared (FT-IR) spectra of Fe-BTC, paclitaxel and Fe-BTC-PTX.

FT-IR was used to investigate possible bond formation between the drug and Fe-BTC. As can be observed in Figure 6, pure Paclitaxel showed all the characteristic peaks in the fingerprint region; 1739 cm^−1^ (ester carbonyl), 1713 cm^−1^ (ketone group), 1657 cm^−1^ (amide group), 1242 cm^−1^ (bending vibrations of C–O–O), 1071 cm^−1^ (ester C–O vibration), 711 cm^−1^ (aromatic C single bond H) [5]. Fe-BTC showed all its characteristic peaks; a broad peak at 3417cm^−1^ assigned to vibration of –OH groups existed in ligands [42], peaks at 1628 and 1576 cm^−1^ attributed to C=O vibrations, peak at 1448 cm^−1^ attributed to C–C vibrations and peak at 1380 cm^−1^ are attributed to C–O group [42,43]. The bands at 761 and 712 cm^−1^ are attributed to the out-of-plane vibrations of C–H groups [42,43]. After paclitaxel adsorption chemical shifts from 3417 to 3389 cm^−1^ and another was appeared at 3438 cm^−1^, a shift from 1576 to 1573 cm^−1^ and from 1448 to 1444 cm^−1^, as well as a small shoulder at 1366 cm^−1^. Also, there is a small peak as shoulder at 1734 cm^−1^ while paclitaxel’s peak at 1242 cm^−1^ was shifted at 1254 cm^–1^ after encapsulation in Fe-BTC. All these changes indicate that some interactions are taking place between PTX and Fe-BTC, probably hydrogen bond or π–π interactions (pi-stacking), which we discuss further in the computational section.

**Figure 7:**
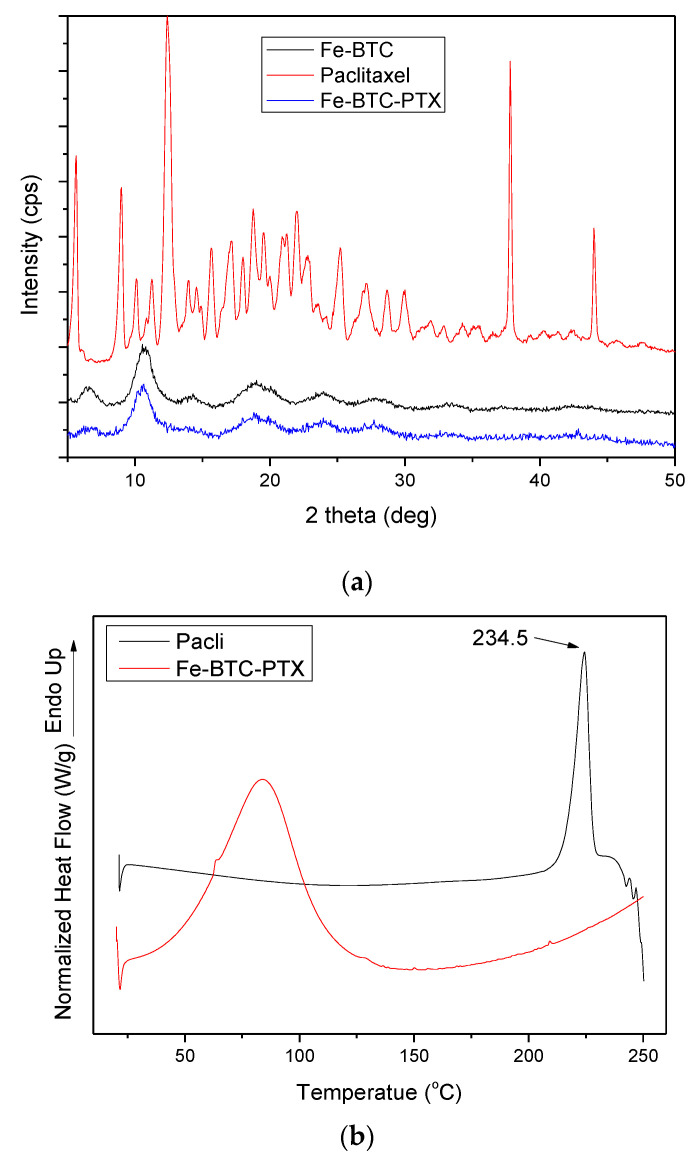
(**a**) XRD patterns of Fe-BTC, Paclitaxel and Fe-BTC-PTX and (**b**) differential scanning calorimetry (DSC) thermographs of Fe-BTC, Paclitaxel and Fe-BTC-PTX.

The crystallinity of paclitaxel, as well as Fe-BTC, was studied before and after the adsorption procedure. As shown in Figure 7a, paclitaxel is a crystalline drug with its main peaks at 5.6°, 8.9°, 12.4°, 37.7° and 43.9° while there are also numerous peaks with lower intensity. Fe-BTC showed a semi-crystalline structure with a broad peak at 10.5° and other five broad peaks with lower intensity at 6.4°, 13.9°, 19.2° and 23.9° [42,44]. In the pattern of Fe-BTC-PTX, i.e., after the adsorption of paclitaxel, the main peak at 10.5° became broader and of lower intensity compared to the neat one, while the intensities of all other peaks appeared very low. No peaks attributed to paclitaxel were observed for the Fe-BTC-PTX nanoformulation’s pattern, leading us to the conclusion that the drug was encapsulated in the amorphous phase. Taking into account the high drug loading of 40% that was estimated by HPLC, this amorphization could be attributed either to the interactions taking place between the drug and Fe-BTC reactive groups, which was already proved from FT-IR spectroscopy, or the incorporation of the drug into the MOF’s pores or their surface and due to their high specific surface area distribution the subsequent alteration of the crystalline structure. It was reported that encapsulating a drug molecule into a MOF leads to amorphous drug confined within the nanoscale pores [45].

DSC measurements were supplementarily performed in order to further verify the aforementioned amorphization. As can be seen in Figure 7b, paclitaxel showed an endothermic peak at 234.5 °C corresponding to its melting point, while its decomposition started after 237 °C, as was also proved by thermogravimetric analysis (TGA) (data not shown). In the Fe-BTC-PTX thermogram there is a broad peak recorded at 90 °C due to the methanol-water evaporation, but no peak was detected for paclitaxel indicating that the drug remains in its amorphous state after its adsorption to Fe-BTC, which is in good agreement with the XRD data.

### 3.3. Characterization of Nanoparticles

Nanoparticles were prepared according to a well-established technique, that of solid-oil-water (s/o/w) as was previously described in Section 2.3.2. The average size and zeta potential of prepared nanoparticles were measured by DLS and the results are shown in Table 2. As can be seen, prepared nanoparticles with neat paclitaxel showed sizes of about 138 nm, a value close to the ones obtained by the same technique and similar aliphatic polyesters used [6,27,46]. This value was increased to 143 nm for nanoparticles prepared with paclitaxel adsorbed to the Fe-BTC, meaning that its incorporation resulted in slightly larger-sized nanoparticles. In both cases, nanoparticles showed narrow polydispersity indexes (PDI). Zeta potential was also estimated, and its values were between −29 and −33 mV meaning that the system was stable and no aggregates were formed. Similar nanoparticles sizes and ζ-potential for mPEG-PCL nanoparticles have also been mentioned recently in literature [47]. 

**Figure 8:**
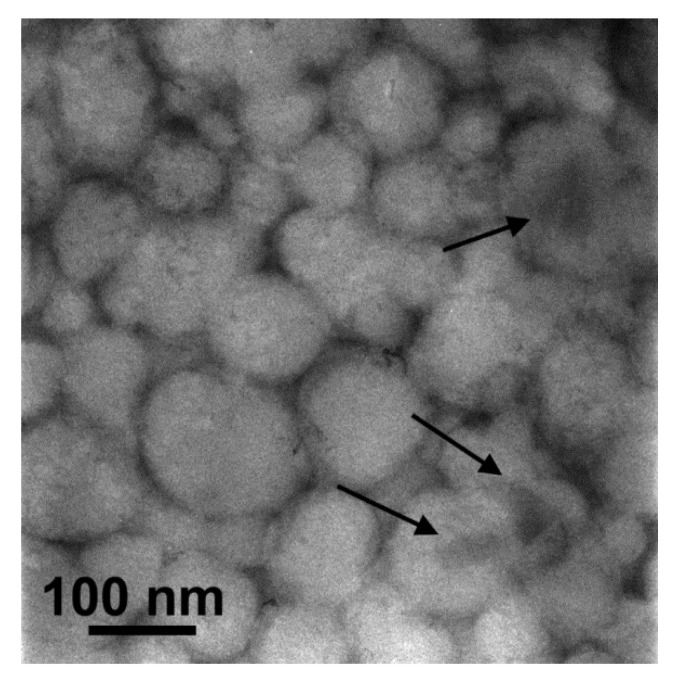
TEM micrograph of the mPEG-PCL-Fe-BTC-PTX nanoparticles showing their spherical shape and the incorporation of taxol loaded Fe-BCT nanoparticles in the polymeric matrix (arrows).

mPEG-PCL nanoparticles containing Fe-BTC MOF loaded with paclitaxel were also examined by TEM imaging. As can be seen from Figure 8, all nanoparticles possess spherical shape with sizes ranging from 80 to 150 nm, that are close to the values found by DLS (Table 2). The darker contrast inside some of them implies the incorporation of PTX-loaded Fe-BTC. Furthermore, no free Fe-BTC nanoparticles, located outside the mPEG-PCL, were observed. This suggests that the majority of the PTX-Fe-BTC nanoparticles have been embedded inside the polymer particles.

**Figure 9:**
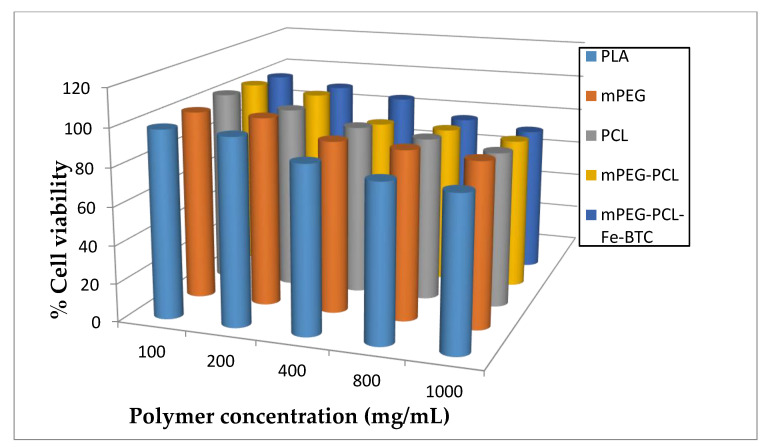
Human umbilical vein endothelial cells (HUVEC) viability after incubation for 24 h for different concentrations of mPEG, PCL, their mPEG-PCL block copolymers and copolymers containing Fe-BTC compared with biocompatible poly(lactic acid) (PLA).

As was found in our previous work, PCL is a biocompatible polyester as well as its copolymers with other aliphatic polyesters [27]. In Figure 9 the HUVEC viability of all prepared materials in comparison with neat mPEG, PCL and PLA after incubation for 24 h is presented. Results showed that the initial mPEG used for synthesis of mPEG-PCL copolymer, as well PCL, are biocompatible polymers since both exhibited low toxicity against HUVEC. The results for the mPEG-PCL copolymer are similar, as expected, since it was extensively used for pharmaceutical applications [48], and also for the composites containing Fe-BTC, which are in total agreement with literature where it is reported that iron (III) based MOFs are non-toxic and have been used as nanocarriers for drugs against cancers and acquired immune deficiency syndrome (AIDs) [49]. Some noticeable cytotoxicity (higher than 20% reduction of cell viability) can be seen in all studied polymers, included PLA, only after exposing the cells at high nanoparticle concentrations, i.e., higher than 800 μg/mL. Based on these results, it is clear that mPEG-PCL and the copolymer containing Fe-BTC are biocompatible and their biocompatibility is similar to that of PLA, a polymer of high biocompatibility which is extensively used in biomedical and pharmaceutical technology [50,51,52].

**Figure 10:**
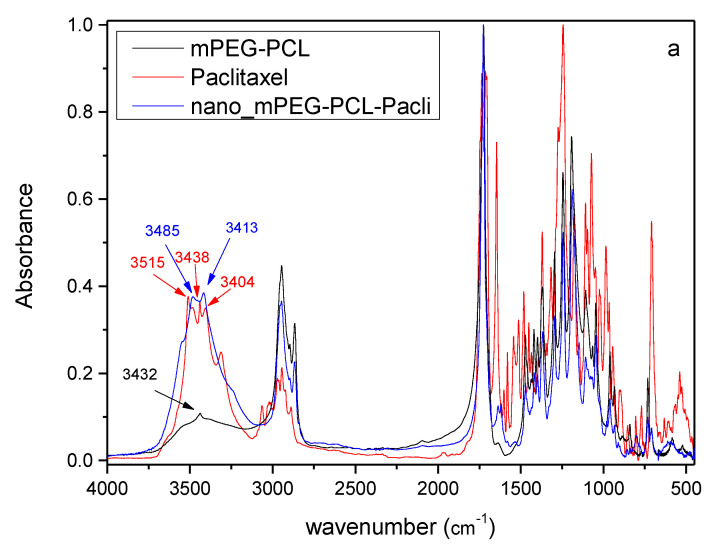

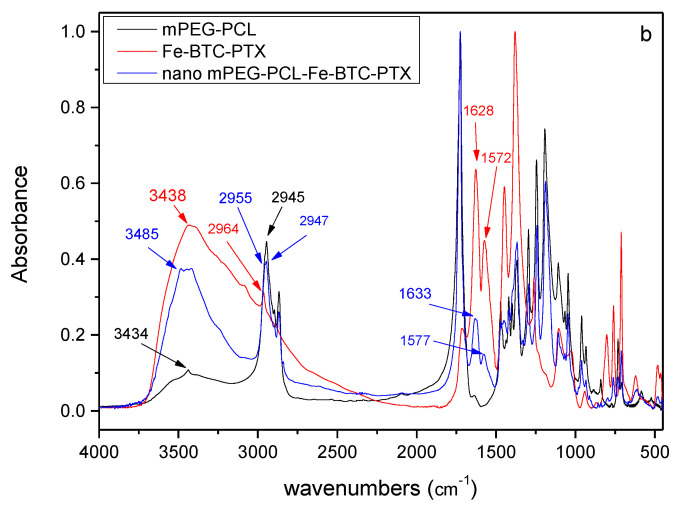
FT-IR spectra of prepared nanoparticles (**a**) with PTX and (**b**) with Fe-BTC-PTX.

FT-IR spectroscopy was again applied in order to evaluate any possible hydrogen bond formation between mPEG-PCL and paclitaxel before and after its adsorption to Fe-BTC. As can be observed in Figure 10a, chemical shifts are observed in the area between 3350 and 3500 cm^−1^, an area corresponding to –OH bonds. In brief, the peaks of paclitaxel at 3515, 3438 and 3413 cm^−1^ were shifted to 3485 and 3413 cm^−1^ showing possible bond formation. Similar are the findings for Fe-BTC-PTX after its incorporation to mPEG-PCL nanoparticles. Chemical shifts are observed from 3438 cm^−1^ of Fe-BTC-PTX and 3434 cm^−1^ of mPEG-PCL to higher wave numbers of 3485 cm^−1^, indicating that possible bond formation took place between the polymer and the absorbed nanoformulation.

**Figure 11:**
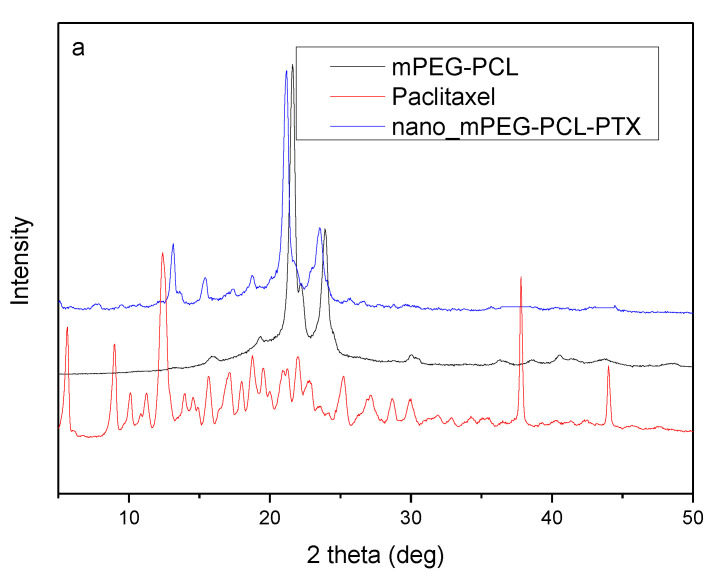

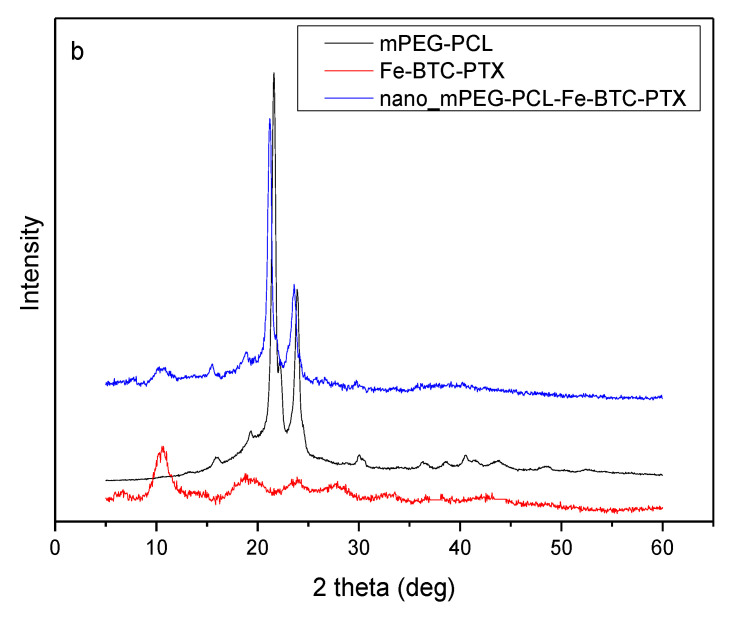
XRD spectra of the prepared nanoparticles (**a**) with PTX and (**b**) with Fe-BTC-PTX.

XRD was used to characterize all prepared nanoparticles. As it can be seen in Figure 11a, nanoparticles with paclitaxel showed peaks at 20.5° and 23° owing to the mPEG-PCL matrix. Some other peaks of lower intensity are also present, revealing some crystallinity due to drug encapsulation. There is a clear peak at 13.17° and some others of low intensity at 9.51°, 7.8° and 5.9°, which are very close to the characteristic peaks of PTX. This is an indication that PTX could be slightly crystallised during its encapsulation in the mPEG-PCL nanoparticles. However, due to the low intensity of these peaks, it is evident that the major part of the drug was encapsulated in its amorphous form. No such results were found after paclitaxel adsorption to Fe-BTC and its further encapsulation into the mPEG-PCL-Fe-BTC-PTX nanoparticles. No peaks were recorded that would be attributed to the drug, whereas the two peaks that appeared at 20.5° and 23° again correspond to the polymer, meaning that after the nanoparticle preparation procedure, paclitaxel remained amorphous in the Fe-BTC framework. This could be attributed to the fine dispersion of the drug in the Fe-BTC, which leads to complete drug amorphization (Figure 7).

**Figure 12:**
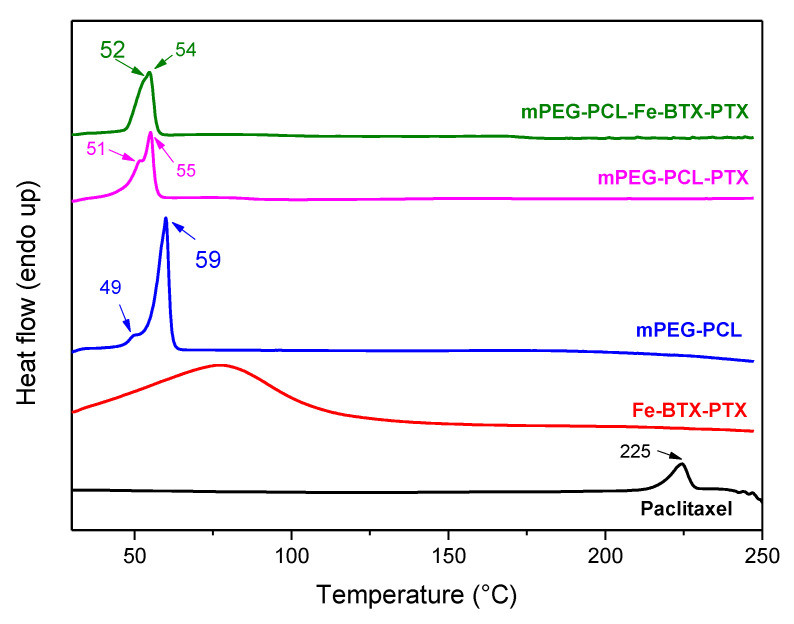
DSC thermogram of the prepared nanoparticles.

In order to better examine the crystallinity of the drug in the nanoparticles, DSC measurements were performed once more. As can be observed in Figure 12, no T_m_ peak of paclitaxel was recorded in any of the samples, meaning that the drug could be in an amorphous form in both nanoparticles. In neat mPEG-PCL, there is a small peak at 49 °C due to the mPEG, which has only a small presence in the copolymer (25 wt%), and one of much higher intensity at 59 °C due to the melting of PCL block. When PTX was incorporated inside the polymer matrix this peak was shifted to 55 °C, indicating that the small molecule of PTX can act as a plasticiser to the polymer matrix. A similar shift was also recorded at the polymer’s T_m_ after incorporation of Fe-BTC-PTX. These shifts could also be attributed to the interactions that take place between the drug and the polymer. However, based on previous studies of ours [8,53], when dealing with polymers of much lower melting point than that of the drug, it is hard, relying only on the DSC results, to conclude whether the drug (in this case PTX) remains in the crystalline or amorphous form inside the nanoparticles. That is because even if the drug is encapsulated in crystalline form, its melting point will not be recorded since this crystalline form dissolves in the melt of the polymer matrix. This could explain the disagreement with the previous XRD data, where at least in the case of mPEG-PCL-PTX formulation, some crystallinity from the drug was observed, but because of the very low melting point of mPEG-PCL, its peak is not recorded in the corresponding DSC thermogram as expected.

HPLC was used to quantify drug content of paclitaxel in nanoparticles and calculate their yield and entrapment efficiency (Table 3). Nanoparticles showed enhanced yield with values appearing to be about 70% for those without Fe-BTC, while just slightly lower is the yield for nanoparticles with Fe-BTC with value of about 65%. Both values are high enough, showing that the procedure is well established. Drug loading is higher for nanoparticles without Fe-BTC reaching 7.5%, a satisfactory value for nanoparticles containing hydrophobic drugs, prepared by this specific method. Nanoparticles with paclitaxel adsorbed onto Fe-BTC were found to contain the drug in about 4%.

### 3.4. Computational Study

#### 3.4.1. Structural Analysis

**Figure 13:**
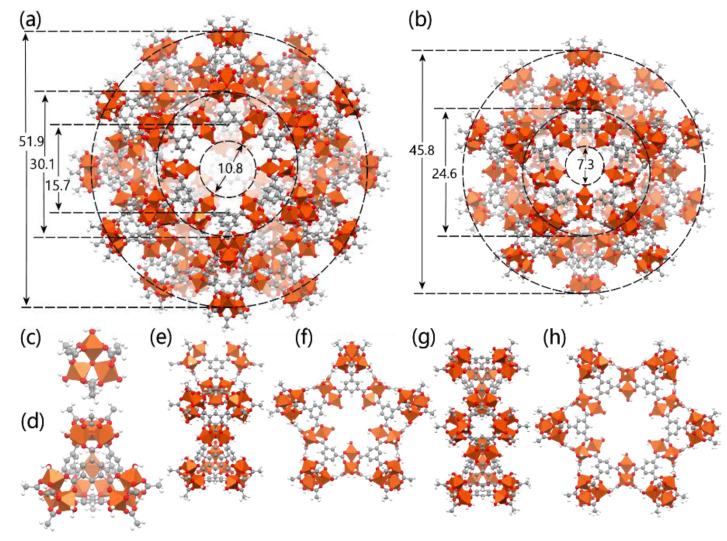
Structural components (acetate models of the building blocks) of Fe-BTC and MIL-100(Fe). (**a**) The large cage denoted here as l-cage, has a pore window diameter of 10.8 Å, cavity diameter of 30.1 Å and an overall size of about 52 Å, (**b**) the small cage denoted here as s-cage, has a pore window diameter of 7.3 Å, cavity diameter of 24.6 Å and an overall size of about 46 Å, (**c**) the iron trimer that is the secondary building unit, (**d**) the super tetrahedron structure, (**e**) side view and (**f**) face view of the isolated five-membered pore window, and (**g**) side view and (**h**) face view of the isolated six-membered pore window (encountered only in l-cage). Structures are optimized at the GFN1-xTB level of theory. Represented structured are not under scale.

The MIL-100(Fe) and Fe-BTC structures consist of similar building blocks with the former material being crystalline [54,55]. The secondary building blocks (SBU) are Fe(III)(*μ*_3_-O) trimers that are linked by BTC forming a super tetrahedron structure. In Figure 13c,d, we show acetate models of the iron trimers and the super tetrahedron structures. The crystalline structure of MIL-100(Fe) consists of linked super tetrahedra forming a canonical network of two type of cages that we denote as s-cage and l-cage. Such cages are expected to form in Fe-BTC as well but in a semi-amorphous configuration; however, there have been reports in the literature that only the smaller, s-cage, structures were identified [56]. The s-cage structure consists a cavity accessible via a total of twelve pore windows each formed by five linked super tetrahedra. The larger, l-cage, structure includes four 6-membered pore windows, along with twelve 5-membered windows. Optimized geometries of the structures are shown in Figure 13 including measured dimensions, along with isolated acetate models of the 6-membered and 5-membered pores windows.

The large cage denoted as l-cage, has a pore window diameter of 10.8 Å, cavity diameter of 30.1 Å and an overall size of about 52 Å, while the small cage denoted here as s-cage, has a pore window diameter of 7.3 Å, cavity diameter of 24.6 Å and an overall size of about 46 Å. The size of Taxol, however, is such (a simplistic triaxial ellipsoidal representation would have principle axes with lengths of 18.2 Å, 15.2 Å, and 7.9 Å) that it can easily reside within either one of the cages to reach the cavities Taxol is required to cross the pore windows. The 5-membered window is marginally too small; however, the 6-membered window can permit traverse of Taxol for suitable orientations. Being semi-amorphic, Fe-BTC should also consist of domains of diverse construction, with larger and irregular cavities. These regions offer an area with which Taxol can interact and accumulate; however, it may be the case that proximity to them be achieved only via 6-membered pore windows or any irregular larger pores. It is thus clear that taxol can enter inside some Fe-BTC pores, albeit not freely, which may be the reason, as was signified by the TEM micrographs, that taxol is mainly located on the surface of Fe-BTC MOFs.

#### 3.4.2. Vibrational Analysis

**Figure 14:**
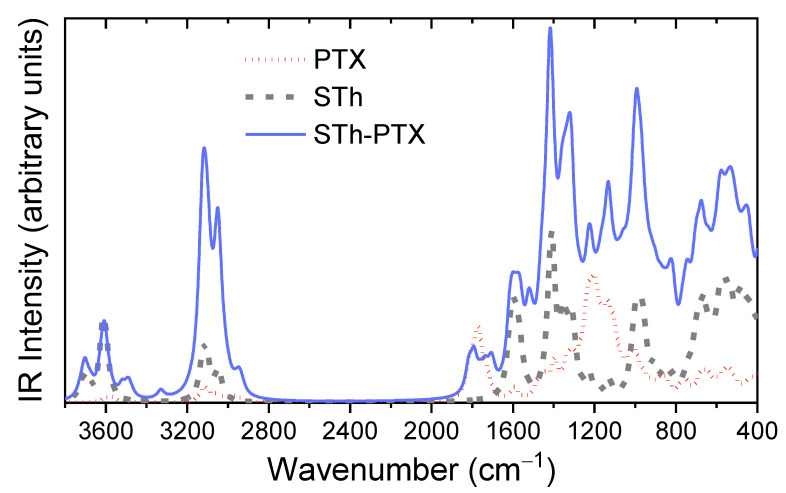
Computed infrared (IR) spectrum of Taxol (PTX) shown by the red dotted line, the super tetrahedron (STh) shown by the gray dashed line, and the interacting super tetrahedron—Taxol system shown by the solid blue line. To be viewed in parallel to Figure 6. Computed at the GFN1-xTB level of theory.

An insight into the interaction between Taxol and Fe-BTC can be obtained by examining the IR spectrum of the interacting system in comparison to the interacting components, as well as to the respective experimental spectra. A direct comparison of the computed and experimental spectra reveals that the computed wavenumbers are overestimated in the large wavenumber region, and are in good agreements in the lower wavenumber range (below 2000 cm^−1^) (Figure 14). The hydroxyl hydrogen vibrations in Taxol are found between 3610–3567 cm^−1^. In the composite Fe-BTC-PTX system in this wavenumber region, the symmetric stretching modes of the coordinated water molecules are found, with the hydroxyl hydrogen vibrations located at the lower end. However, vibrations of water hydrogen atoms that are interacting by hydrogen bonding with PTX carbonyl have smaller wavenumbers, between 3520–3490 cm^−1^. In the computed spectra, this is identified by the formation of a peak around 3500 cm^−1^ in the composite system spectrum that is not found on either of the distinct systems and has a prospect of being a signature mode of PTC-Fe-BTC interaction. The resolution of the experimental spectra in this region does not allow for a clear identification of this effect and may be obscured between 3200–3350 cm^–1^, thus limiting its usefulness as a signature mode. Between 3183–2930 cm^−1^ the vibrational modes are found to correspond to hydrogen atoms of the benzenes, asymmetric modes in methyl groups followed by symmetric modes in methyl groups. Directly after the gapped region the vibrational modes of the carbonyl double bonds are found, from 1816–1702 cm^−1^. This is attributed to the peaks around 1734 cm^−1^ in the experimental spectrum. The C–C stretching mode of the benzene groups of taxol is located in the region 1697–1586 cm^−1^. Within this region in the super tetrahedron structure and specifically in the range 1553–1631 cm^−1^ lies the C–C stretching mode of the BTC benzene groups mixed with the antisymmetric stretching of the carboxyl linking oxygen atoms. No shifts are noted for these modes in the composite system. The symmetric stretching of the carboxyl linking oxygen atoms of the super tetrahedron are between 1359–1314 cm^−1^ while in the composite system the range remains only slightly shifted at 1363–1332 cm^−1^ and should correspond to the experimental peak at 1380 cm^−1^. The intense peaks of PTX at 1198 cm^−1^ and 1191 cm^−1^ correspond to C–O stretching between the bridging oxygen atom and the carbonyl carbon atom. These modes are identified at 1214 cm^−1^ and 1281 cm^−1^ and remain intense in the composite system and slightly shifted. In the experimental spectrum of the composite system (see Fe-BTC-PTX in Figure 6) these modes are expected to correspond to the peak at 1262 cm^−1^. Considering that Fe-BTC exhibits only weak peaks in the near vicinity of that wavenumber, we note this shifted and intense peak of the composite systems as the best signature peak that indicates (a) the presence of PTX and (b) the PTX interaction with Fe-BTC.

#### 3.4.3. Intermolecular Interactions

Given the size of Taxol relative to the components of Fe-BTC, we examined configurations relative to the super tetrahedron. The two strongest interacting configurations differ by 0.83 kcal/mol. The interaction energy has been calculated using,

(7) $$E_{\mathrm{int}}\;=\;E_{\mathrm{STh}+\mathrm{PTX}}\;-\;\left(E_{\mathrm{STh}}\;+\;E_{\mathrm{PTX}}\right)$$

where $E_{\mathrm{STh}+\mathrm{PT}X}$ is the energy of the super tetrahedron and Taxol interacting system, $E_{\mathrm{PTX}}$ is the energy of the Taxol molecule, and $E_{\mathrm{STh}}$ is the energy of the super tetrahedron that carries a charge of +4e (i.e., +e per iron trimer) in a spin state with multiplicity of 17.

**Figure 15:**
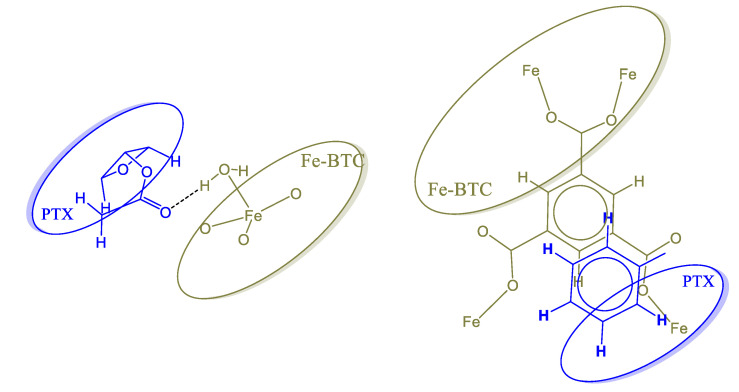
PTX-Fe-BTC primary interaction sites. (**Left**) Hydrogen bonding between carbonyl oxygen atom and coordinated water (two instances identified). (**Right**) Parallel displaced pi-stacking between benzene groups of PTX and BTC.

The strongest interacting configuration exhibits three interaction sites; two Taxol carbonyl oxygen atoms form hydrogen bonds with coordinated water molecules with lengths of 1.92 Å and 1.95 Å, as well as parallel displaced pi-stacking between benzene of BTC at a distance of 3.21 Å and displacement of 2.4 Å (Figure 15). A secondary interaction site is between a Taxol bridging oxygen and a coordinated water hydrogen, with a distance of 2.25 Å. The cumulated interaction is strong, computed at 80 kcal/mol. The high interaction energy value of PTX with the super tetrahedron structure suggests that PTX molecules can attach strongly and may block the pore windows preventing any further entrance of PTX to the cage cavity. As such and in spite of having the same fundamental building blocks, Fe-BTC comes out as being more suitable than MIL-100(Fe) due to its amorphous construction that can include larger cavities and pore windows that are not subject to the aforementioned clogging.

### 3.5. In Vitro Drug Release

**Figure 16:**
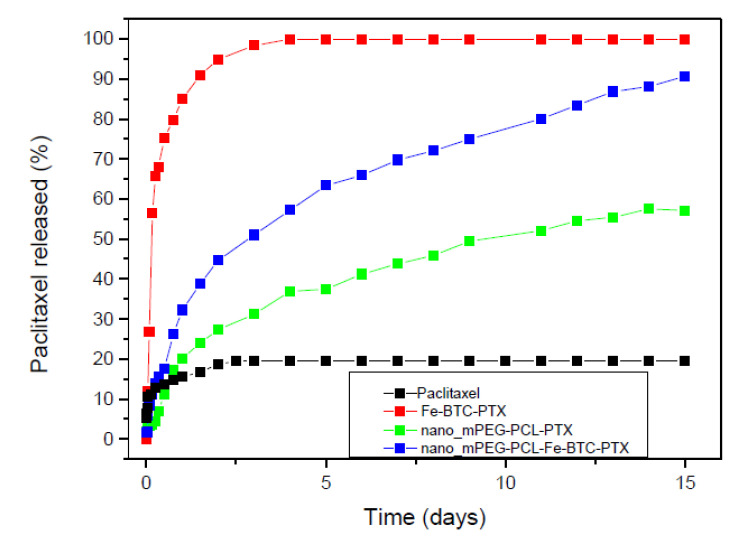
Dissolution study in vitro of paclitaxel, paclitaxel adsorbed to Fe-BTC and nanoparticle systems.

Figure 16 demonstrates the comparative in vitro dissolution profiles of neat paclitaxel, PTX adsorbed on Fe-BTC, and PTX (or PTX-Fe-BTC) encapsulated in the mPEG-PCL nano-formulations. As illustrated, the dissolution rate of neat paclitaxel reached its maximum of about 13% within the first 3 days with no further increase observed thereafter [5,6,57,58]. This is due to its high hydrophobicity and its crystalline structure. Its absorption onto Fe-BTC was found to significantly improve its release rate, achieving the maximum value of nearly 100% in the same time period. More specifically, the drug release followed a two-stage release process. During the first two days, a dissolution rate of 85–90% was observed, probably owing to the drug adsorbed onto the MOF’s surface and its consequent drug amorphization led to an enhanced dissolution. At a further second stage up until the third day of the study, a slight increase equal to 95–97% can be seen, probably due to the paclitaxel incorporated into the pores of Fe-BTC. A similar two-phase release profile was also mentioned regarding the delivery of the poorly water-soluble drug Flurbiprofen using different biocompatible MOFs [59]. Yet, it seems more than evident that the integration of the Fe-BTC in the drug delivery system leads to a substantially higher dissolution rate. Comparable results were also reported in a previous work of ours, where solid dispersions of fumed silica nanoparticles containing the poorly water-soluble drug tibolone were prepared [60]. The dissolution enhancement was in that case due to the finer distribution of tibolone onto the large specific area of SiO_2_ nanoparticles, which resulted in the drug’s amorphization [61,62].

**Figure 17:**
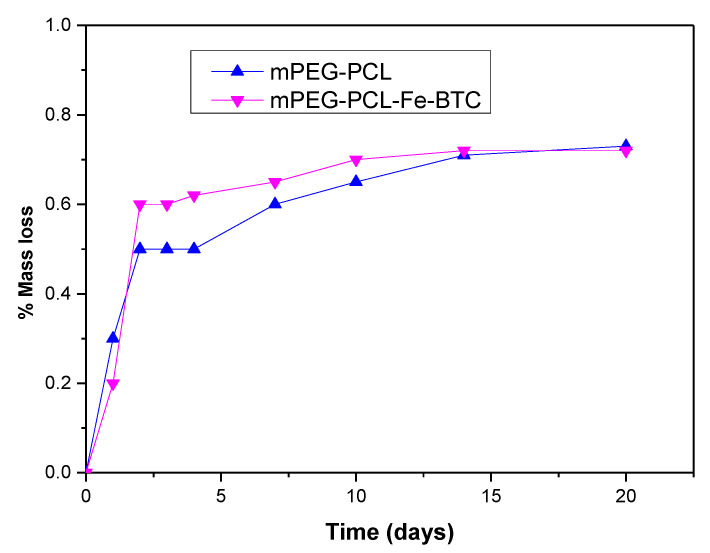
Mass loss of mPEG-PCL copolymer and its mPEG-PCL-Fe-BTC nanocomposite during hydrolysis in phosphate buffer (pH = 7.4, *T* = 37 ± 0.5 °C).

A multi-phasic drug release was also observed for nanoparticles prepared by mPEG-PCL. In the initial stage lasting 2–3 days, a release of about 20–25% was observed corresponding to a burst release effect. This is due to the drug being absorbed on nanoparticles surface or very close to it [8,63,64]. It could also be attributed to the possible hydrolysis of the copolymer. However, during the hydrolysis test in pH 7.4 at 37 °C, which are similar conditions with that of the dissolution studies, the mass loss is less than 1 wt% (Figure 17). PCL is a polymer well known for its high degree of crystallinity and the inherent slow hydrolytic degradation rate. Even after the addition of the hydrophilic mPEG, its hydrolysis rate remains too low and our results are in agreement with that reported recently for similar copolymers [65]. Higher degradation rates have been mentioned only in alkaline or acidic conditions [66]. Therefore, it is clear that the initial burst release of paclitaxel is due to the drug located on the nanoparticles’ surface and that this immediate release is not attributed to the polymer hydrolysis. After the third day, paclitaxel continued to be released from mPEG-PCL nanoparticles almost steadily and linearly. This is an indication that diffusion perhaps controls drug release at this stage. Comparing the release rate of PTX from mPEG-PCL with the rate of neat PTX, it can be seen that it is much higher in the case of nanoparticles and this can be attributed to the partial drug amorphization inside the polymer matrix. It is well known that amorphous drugs have much higher solubility than crystalline ones [67,68]. A quite similar behaviour can be also seen in mPEG-PCL nanoparticles containing Fe-BTC-PTX. There is an initial burst release up to day 3 and, thereafter, there is a continuous and steadily release till day 15. Compared to the mPEG-PCL nanoparticles, it is evident that the burst release is much higher in nanocomposites and this can be attributed to the complete amorphization of PTX after its incorporation in Fe-BTC. For this reason, the release profile is much better in mPEG-PCL-Fe-BTC-PTX nanocomposites than neat PTX and reaches almost 90% at day 15. Furthermore, it is clear that the release rate is much higher even from neat mPEG-PCL-PTX nanoparticles. Drug release from nanoparticles depends on several factors, such as the particle size of the nanoparticles, the molecular weight and degree of crystallinity of used polymer, and its melting point matrix [69]. In the present study, nanoparticle size is almost identical and the polymer matrix is also the same. Thus, these factors should not affect the release behaviour of PTX. Again hydrolytic degradation could also affect the drug release. However, there are no significant differences in mass loss between mPEG-PCL and mPEG-PCL-Fe-BTC (Figure 17). Drug crystallinity is another important factor and, as was found from XRD patterns, PTX is encapsulated in partially amorphous form in the mPEG-PCL matrix and in completely amorphous form in mPEG-PCL-Fe-BTC-PTX. Consequently, drug amorphization plays the most important role and affects the release profiles of the different nanoparticles here.

### 3.6. Modeling of the Release Kinetics

In this section an attempt to describe the experimental drug release kinetics considering some physical mechanisms is presented. There are actually four different release curves with at least three different released mechanisms. The release mechanism of the neat drug is dissolution with the rate determined by a combination of a dissolution reaction (transition of drug from the solid to liquid phase) and mass transfer in a liquid. No attempt to model this process is presented here since the focus is on the controlled release. The second release curve refers to drug release from Fe-BTC nanoparticles. The loading here is achieved through an adsorption process and thus the release mechanism is desorption. This means that the adsorbed molecules in the polymer matrix desorb and then diffuse through the polymer matrix up to the bulk liquid. The small pore size of Fe-BTC (see Table 1) denotes that the surface diffusion is the dominant desorption mechanism. The term surface diffusion implies that the drug diffuses in its adsorbed state along the pore walls and not in a dissolved state in the fluid filling the pores. The kinetics of such a desorption process is typically described through the solution of the transient diffusion equation in spherical coordinates (assuming a spherical particle shape). This solution leads to the following equation for the calculation of the percentage of drug release *R* (i.e., quantity presented in Figure 16) [70]:

(8) $$R=100(1-\frac{6}{\pi^{2}}\sum_{i=1}^{\infty }\frac{1}{i^{2}}\exp(-Di^{2}\pi^{2}t/r^{2}))$$

where *D* is the surface diffusion coefficient and *r* is the Fe-BTC nanoparticle radius. A simply larger time approximation of the above series solution results from keeping only the first exponential of the series and modifying its parameters. The main difference from the exact solution is its inability to predict the so-called initial diffusion burst.

Prior to the fitting procedure, it is essential to define a measurement of deviation between experimental and model release data. Let *N* is the number of non-trivial (*R* < 99) data points. The fitting quality is assessed through the following root of mean squared deviations:

(9) $$S={\left[\sum_{i=1}^{N}\frac{{\left(R_{\mathrm{mod}el}-R_{\exp}\right)}^{2}}{N}\right]}^{1/2}$$

The fitting attempt using the single term approximation and the complete series solution (8) led to values of *S* equal to 8.3 and 4.8 respectively. These values are too large denoting that the models are inappropriate to describe the data. Another approach is followed: Two exponential terms are retained in Equation (8) but with both exponents being fitting parameters. The model equation now has the form:

(10) $$R=100[\varphi (1-\exp(-K_{1}t))+(1-\varphi )(1-\exp(-K_{2}t))]$$

**Figure 18:**
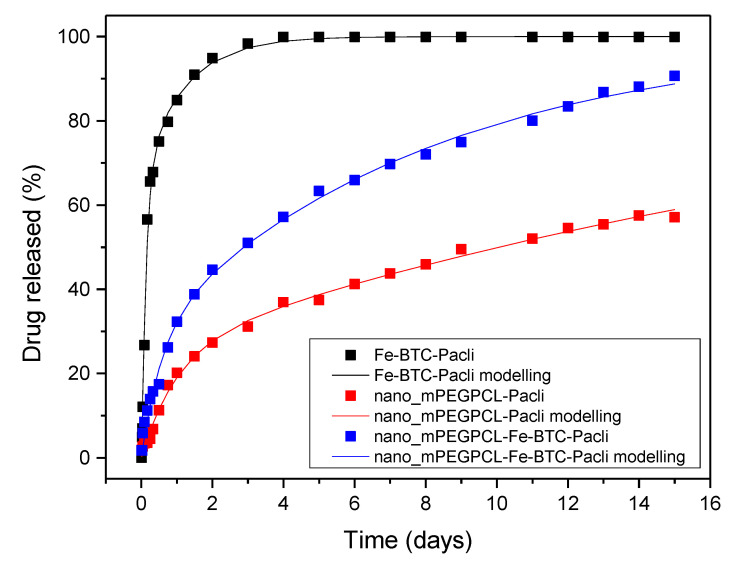
Comparison between experimental (markers) and model (solid lines) drug-release evolution curves.

Such a model implies that there are two different populations of diffusing molecules with fractions *φ* and 1 − *φ* and two different diffusion coefficients. The diffusion coefficients *D_i_* (*i* = 1,2) are related to the fitting constants K_i_ through the relation (based on linear driving force approximation) *K_i_* = 15*D_i_*/*r*^2^ [71]. The fitting procedure led to a value *S* = 2.8 which is comparable to the scatter of the data. The fitting constants are *φ* = 0.665, *D*_1_ = 6∙10^−22^ m^2^/s, *D*_2_ = 6.6∙10^−23^ m^2^/s. The values of the diffusion coefficients are extremely low but this is not unexpected considering that the drug molecule size and the pore size are comparable inhibiting the motion of the drug along the pores. There is an order of magnitude difference between the two populations’ diffusion coefficients. This bimodality may be induced by any kind of non-uniformity either structural of the polymer matrix or spatial of the drug distribution in the matrix. In particular it is quite compatible to the finding of the structural analysis that taxol enters the pores but mainly accumulates at the surface of Fe-BTC nanoparticles. The comparison between experimental and model release curve appears in Figure 18.

The next step is the description of the release kinetics from mPEG-PCL and composite Fe-BTC in mPEG-PCL nanoparticles. The standard models for drug release from polymer matrices [72] cannot describe the results. In addition, the shape of the curves is not compatible with the solution of diffusion equation appearing in relation (8). However, from the physical point of view is expected that the dominant release mechanism is the drug diffusion through mPEG-PCL matrix. A small contribution of polymer hydrolysis is expected but as hydrolysis is certainly much smaller (less than 1%) than the enzymatic hydrolysis, as presented in Figure 17, it hardly affects the release behaviour. The next step is to invoke the bimodal exponential equation used above (i.e., Equation (10)). This equation is fitted extremely well to the experimental data. The values of S are 1.1 and 1.65 for the simple and composite nanoparticles respectively. The comparison between model curves and experimental data appears again in Figure 18.

The fitting parameters are *φ* = 0.254, *Κ*_1_ = 1 d^−1^, *K*_2_ = 0.04 d^−1^ for the mPEG-PCL nanoparticles and *φ* = 0.29, *Κ*_1_ = 1.74 d^−1^, *K*_2_ = 0.123 d^−1^ for the mPEG-PCL-Fe-BTC nanoparticles. The parameters K_1_, K_2_ can be transformed to diffusion coefficients through the relation *D* = *Kr*^2^/15 where r refers to the radius of the corresponding nanoparticle. The results are *D*_1_ = 3.7∙10^−21^ m^2^/s, *D*_2_ = 1.46∙10^−22^ m^2^/s for mPEG-PCL and *D*_1_ = 6.9∙10^−21^ m^2^/s, *D*_2_ = 4.84∙10^−22^ m^2^/s for mPEG-PCL-Fe-BTC. The release curves suggest that a complete (100%) release is approached in the case of mPEG-PCL-Fe-BTC nanoparticles. It is expected from a physical point of view that this will be also the case for mPEG-PCL nanoparticles and the observed deficiency is of purely kinetic origin. This picture is compatible with the predictions of the fitted model. For both types of nanoparticles, the fitted model indicates that a fraction of the drug (25–30% of the total drug) undergoes fast diffusion whereas the rest undergoes slow diffusion. The diffusion coefficients of fast mode appear to be an order of magnitude larger than those of the slow mode. It is interesting that the addition of the drug through Fe-BTC increases the diffusion coefficients by 86% (fast mode) and 230% (slow mode) compared to its direct addition in mPEG-PCL nanoparticles. A possible explanation for this is that the Fe-BTC nanoparticles are distributed in the mPEG-PCL nanoparticles in an advantageous way with respect to a single drug (i.e., closer to the particle surface). In addition, a possible small contribution to the “fast” diffusion mode may come from the polymer matrix hydrolysis. It is noted that for such small values of diffusion coefficients not only steric interactions but also electrostatic ones between drug and polymer matrix must be essential. However those interactions are influenced by variables such as pH and the ionic strength of the liquid [73,74] which are expected to affect in this way the release kinetics.

## 4. Conclusions

In this study, novel therapeutic nanoparticles for the delivery of the paclitaxel anticancer drug were prepared. The novelty here lies in the use of Fe-BTC, a MOF with high porosity, in the development of a drug-delivery system for the first time. Paclitaxel was adsorbed onto the Fe-BTC and the resulting nanocomposite was encapsulated into mPEG-PCL nanoparticles. It was found that the adsorption of the drug into the Fe-BTC network led to its amorphization, while the amount entrapped reached a 40% *w*/*w*, a value relatively high compared to other MOFs used in literature. Drug amorphization was due to the interactions taking place between Fe-BTC and taxol, proved by FT-IR spectroscopy, as well as due to the drug adsorption and dispersion into a high specific area of used MOF. Paclitaxel’s release was enhanced after its incorporation to Fe-BTC, whereas its nanoencapsulation into mPEG-PCL amphiphilic copolymers was shown to control its release. In compliance with our studies, it is clear that the absorption of paclitaxel on Fe-BTC resulted in substantial enhancement of its dissolution and also due to its complete amorphization, the drug release of mPEG-PCL-Fe-BTC nanoparticles is much higher compared to that of mPEG-PCL nanoparticles. Analysis of the release data suggests that drug diffusion seemed to be the main factor affecting the release behaviour. Computations reveal that Paclitaxel attaches effectively to Fe-BTC building blocks. The relatively large size of the paclitaxel molecule limits its diffusion through the crystalline regions of Fe-BTC; however, the semi-amorphous structure of Fe-BTC renders it a more suitable material than its crystalline counterpart MIL-100(Fe). According to these, it is clear that taxol absorption into Fe-BTC MOFs and their additional nanoencapsulation in mPEG-PCL nanoparticles with sizes of 80–150 nm is an appropriate strategy for dissolution enhancement of taxol and also to provide formulations with a controlled release.

## Figures and Tables

**Table 1:** Porosity data of Fe metal ions and 1,3,5-benzenetricarboxylate (Fe-BTC) and Fe-BTC with adsorbed paclitaxel (Fe-BTC-PTX).

Sample	SSA ^a^ (m^2^/g)	Total Pore Volume ^b^ (cc/g)	Micropore Volume ^c^ (cc/g)	Micropore Area (m^2^/g)	Average Pore Diameter ^d^ (nm)
Fe-BTC	1360	0.798	0.257	658.71	1.2
Fe-BTC-PTX	826	0.428	0.195	470.74	1.2

^a^ Multi-point BET-method using N_2_ adsorption data at −196 °C. ^b^ At *P*/*P_0_* = 0.99. ^c^ t-plot method. ^d^ Average mesopore diameter by BJH analysis using N_2_ adsorption data.

**Table 2:** Average size and ζ-potential values for nanoparticle systems obtained by dynamic light scattering.

Sample (Nanoparticles)	Average Size (nm)	Polydispersity Index (PDI)	Z-Potential (mV)
nano mPEG-PCL-PTX	138 ± 0.54	0.24 ± 0.06	−29.58 ± 0.04
nano mPEG-PCL-Fe-BTX-PTX	143 ± 0.59	0.23 ± 0.07	−32.49 ± 0.08

**Table 3:** Nanoparticles yield, drug loading and entrapment efficiency of nanoparticle systems.

Sample	Nanoparticles Yield (%)	Drug Loading (%)	Entrapment Efficiency (%)
mPEG-PCL-PTX	70.22 ± 0.09	7.48 ± 0.17	74.79 ± 0.07
mPEG-PCL-Fe-BTX-PTX	64.79 ± 0.15	3.92 ± 0.16	10.00 ± 0.03
